# Putative Biomarkers and Targets of Estrogen Receptor Negative Human Breast Cancer

**DOI:** 10.3390/ijms12074504

**Published:** 2011-07-13

**Authors:** Ziad J. Sahab, Yan-Gao Man, Stephen W. Byers, Qing-Xiang A. Sang

**Affiliations:** 1 Department of Oncology, Lombardi Comprehensive Cancer Center, Georgetown University Medical Center, Washington, DC 20007, USA; E-Mail: byerss@georgetown.edu; 2 Diagnostic and Translational Research Center, Henry Jackson Foundation for the Advancement of Military Medicine, Gaithersburg, MD 20789, USA; E-Mail: ymann@hjfresearch.org; 3 Jilin University, Changchun 130012, China; 4 Department of Chemistry and Biochemistry and Institute of Molecular Biophysics, Florida State University, 102 Varsity Way, Tallahassee, FL 32306, USA; E-Mail: qxsang@chem.fsu.edu

**Keywords:** biomarker, breast cancer, chromatography, DCIS, electrophoresis, estrogen receptor, metastasis, proteomics

## Abstract

Breast cancer is a progressive and potentially fatal disease that affects women of all ages. Like all progressive diseases, early and reliable diagnosis is the key for successful treatment and annihilation. Biomarkers serve as indicators of pathological, physiological, or pharmacological processes. Her2/neu, CA15.3, estrogen receptor (ER), progesterone receptor (PR), and cytokeratins are biomarkers that have been approved by the Food and Drug Administration for disease diagnosis, prognosis, and therapy selection. The structural and functional complexity of protein biomarkers and the heterogeneity of the breast cancer pathology present challenges to the scientific community. Here we review estrogen receptor-related putative breast cancer biomarkers, including those of putative breast cancer stem cells, a minor population of estrogen receptor negative tumor cells that retain the stem cell property of self-renewal. We also review a few promising cytoskeleton targets for ER alpha negative breast cancer.

## 1. Introduction

Cancer cells frequently exhibit unique gene expression profiles resulting not only in their limitless replication, but also in their ability to actively attack other tissues, recruit the collaborating cells necessary for sustained angiogenesis, and afford them protection from the host immune system [1]. Consequently, on the molecular level, tumors are never silent, but are constantly signaling their presence through the release of a diverse range of enzymes, modulators, and mediators [2]. Breast cancer is a heterogeneous disease. Some breast cancer cells lose their ability to express ERα, among other proteins. The resulting disease is a therapy-resistant cancer. To identify human breast cancer biomarkers between ERα(+) and ERα(−) breast tumors, tissues were microdissected and differential protein expression by adjacent tissues was identified [3], microdissected breast tissues composed of either normal ductal epithelium or ductal epithelium containing Ductal Carcinoma *in situ* (DCIS) lesions were also compared [4]. The heterogeneity of breast cancer architecture is currently hindering proteomics research in this area [5]. Promising new biomarker identification methodologies are under way like lectin glycoarray technology [6], microfluidic-based biosensors [7], lectinomics [8], gold nanoparticles [9–11], enrichment of low-abundance proteins [12–20], and dye-doped silica nanoparticle labels [21].

## 2. Estrogen Receptors

Estrogens play a major role in the development of sexual glands and the reproductive cycle [22], with their biological effects mediated through the estrogen receptor (ER). ERα, cloned in 1986 [23,24], was believed to be the sole form of this receptor until 1996, when a second ER, called ER-β, was also cloned [25,26]. Since that time, five ERβ isoforms (ERβ1 through ERβ5) have been cloned and characterized, and their nucleotide sequences are consistent with the incorporation of different exons [27]. The exact roles of ERα and ERβ in breast cancer are still unknown, though it has been reported that estrogens are involved in the promotion of human breast cancer, possibly by way of their mitogenic activity. ERα and ERβ have structural domains that are not conserved [26] and have different transcriptional activity [28] and ligand binding affinity [29]. ERβ requires higher levels of estrogens for activation than does ERα and acts as a transdominant inhibitor of ERα in near-saturating hormone levels [30]. Different forms of the ER are therefore likely to mediate signal transduction in very different fashions, and understanding the role of each ER in the pathogenesis of breast cancer is vital in the development of estrogens for use in long-term hormone replacement regimens that do not promote breast cancer [31]. Studies performed with mice indicate that ERα mediates the major proliferative effects of estrogen, as ERα knockout mice exhibit rudimentary mammary glands and infertility [32,33]. In contrast to this finding, ERβ knockout mice showed normal mammary gland development, but significantly reduced fertility [34]. These studies suggest distinct but overlapping biological actions for these two receptors.

Several *in vitro* studies have been performed to study the effects of these two ERs in human breast cancer cells. Studies with MCF-7 breast cancer cells, which express ERα, revealed that estradiol stimulates proliferation in these cells [35]. Additional studies with the MCF-7 cell line revealed cessation of proliferation when the ERα gene was knocked out, and a resumption of proliferation when the ERα gene was reintroduced [36]. A recent study utilizing ERβ-transfected MCF-7 cells showed inhibition of proliferation *in vitro* and tumor formation *in vivo* in a nude mouse xenograft model in response to estradiol [31]. Studies performed with cervical cancer-derived HeLa cells indicate that estrogens activate cyclin D1 when complexed with ERα. However, the expression of cyclin D1, a major regulator for entry into the proliferative stage of the cell cycle, is inhibited in the presence of ERβ [37]. *In vitro* studies using the breast cancer cell line T47D have shown reduced estradiol-stimulated proliferation when the expression of ERβ mRNA equals that of ERα. This reduction in proliferation correlates with a decrease in proliferation-associated cell cycle components such as cyclin E, Cdc25A, and Cdk2 [38]. Additional studies utilizing the MDA-MB-231 breast cancer cell line have shown that ERα and ERβ are capable of reversing the invasive phenotype of this breast cancer cell line by inhibiting migration and invasion [39]. Combined, these studies suggest that ERα and ERβ may have opposing effects in terms of breast cancer cell proliferation, but similar effects in terms of *in vitro* inhibition of migration and invasion.

Immunohistochemical staining for ERβ in normal human breast tissues, DCIS, invasive cancers, and lymph node metastases has revealed a gradual reduction in ERβ expression during the transition from normal tissue to pre-invasive lesions to invasive cancers, with ERβ completely absent in 21% of the invasive cancers studied [40]. Another study utilizing similar techniques revealed that the percentage of cells positive for ERβ was high in normal mammary glands and non-proliferative benign breast disease, but decreased significantly in proliferative benign breast disease and carcinoma *in situ*. The ratio between ERβ and ERα was high in normal glands, but decreased significantly in proliferative lesions [41]. These results are in agreement with results obtained using *in situ* hybridization to investigate mRNA levels of ERβ in normal mammary, benign breast disease, breast cancer, and metastatic lymph nodes [42]. *In situ* hybridization revealed that ERβ expression was significantly decreased in breast cancer and metastatic lymph node tissues when compared with normal mammary and benign breast disease tissues. All of these results suggest that ERβ might exert a protective effect against the mitogenic activity of estrogens mediated by ERα, and may therefore function as a tumor suppressor, as the loss of ERβ expression seems to correlate with the progression of breast carcinomas.

A small fraction of *in situ* ERα negative breast tumor cell clusters showed signs of stromal and vascular invasion but lacked many differentiation markers, suggesting that these clusters may contain mutated stem cells [43]. Cancer stem cells are a minor population of tumor cells that retain the stem cell property of self-renewal. However, pathways regulating this process in normal stem cells are deregulated in cancer stem cells, leading to the continuous expansion of self-renewing cancer cells and new tumor cluster formation [44]. Targeting cancer stem cells may improve the effectiveness of cancer therapies [45]. Although beyond the scope of this review, it is important to note that ER negative breast cancer cells can later revert back to ER positive cancer following trastuzumab and chemotherapy treatment, except for triple negative breast cancers [46], and putative estrogen receptor positive breast cancer stem cells have been identified [47].

## 3. Putative ERα Breast Cancer Biomarkers

Differential protein expression between ER(+) and ER(−) tissues has been investigated [43,48–54]. Loss of TIP30 enhances the activation of Akt signaling, leading to the development of ER+/PR− mammary tumors [55]. The key driver of the proliferation of ERα(+) is the high expression of microRNA miR-375 [56]. Overexpression of HIF1 in ERα(+) cells cooperates with ER and hypoxia to promote breast cancer progression [57,58]. Contrary to ERα(−) breast tumors, ERα(+) cells have low levels of 611-CTF, a Her2 C-terminal fragment that induces resistance to anti-estrogen therapies [59]. The highest levels of cell proliferation have been observed in invasive carcinomas with increased ERα(+) expression [60]. Levels of ERα(+) are regulated by immunophillin FKBPL, an estrogen receptor gene. Cells expressing FKBPL are more sensitive to anti-estrogen therapies [61]. Ronneberg *et al* have conducted a study to analyze gene methylation and its correlation with ER status [62]. They found 171 CpG sites representing 151 Entrez Gene IDs that were differentially methylated between ERα(+) and ERα(−) breast cancers with the following CpG sites hypermethylated in ER+ tumors: *STAT5A*, *WNT1*, *DAPK1*, *ALPL*, *IFNGR2*, *IGFBP7*, *ST6GAL1* and *TMEFF1*.

CXCR4 overexpression has been shown to promote estrogen independence *in vivo* [63]. Inhibiting telomerase activity has shown efficiency for treating ERα(−) cells [64]. Estrogen receptor negative breast cancers have been associated with focal myoepithelial disruption, a lack of expression of tumor suppressors, and a higher rate of cell proliferation [48,65–71]. Furthermore, the vast majority of cell budding in breast cancer ducts is driven by ERα(−) cells [49]. BP1 was found to be overexpressed in ERα(−) tumors and results in significantly enhanced cell proliferation and metastatic potential [72]. A recent study identified differential protein profiles between ERα(+) and ERα(−) cells microdissected from the same duct [3]. This study showed that ERα(−) cells express lower levels of: (1) superoxide dismutase, a protein that plays a key anti-oxidant role in the cell by converting superoxide radical to hydrogen peroxide and oxygen [73]; (2) RalA binding protein, a Ras-related small GTPase that plays a major role in intracellular membrane trafficking, as well as tumorigenesis, invasion, and metastasis [74,75]; (3) galectin-1, a protein that induces apoptosis in breast cancer cells by blocking the cell cycle at the S/G2 transition [76]; (4) uridine phosphorylase 2, a protein that catalyzes the phosphorolysis of uridine to uracil and is involved in fluoropyrimidine metabolism, playing a role in the intracellular activation of 5-fluorouracil [77]; (5) cellular retinoic acid-binding protein 1 which regulates retinoic acid activity by increasing its degradation rate by enhancing the production of RA metabolizing enzymes [78]; (6) S100 calcium binding protein A11, involved in tumorigenesis [79], although it has been shown to be upregulated in some cancers [80]; and (7) nucleoside diphosphate kinase A or non-metastasis protein 23-H1 (nm23-H1), a metastasis suppressor gene in which restoration reduces metastasis of breast cancer [81,82]. ERα(−) microdissected cells expressed higher levels of Rho GDP-dissociation inhibitor 1 alpha, a cellular protein that controls the cellular distribution and activity of Rho GTPases and reported to promote the resistance of cancer cells to drug-induced toxicity, thus playing an anti-apoptotic role [83–85]. The collective role of the alterations of protein expression in ERα(−) cells may be to promote a more malignant phenotype than adjacent ERα(+) cells, including a decreased ability to undergo apoptosis and differentiation and an increased potential to damage DNA, metastasize, and resist chemotherapy. A percentage of ERα(−) breast cancer cells are composed of breast cancer stem cells, the most aggressive type of breast cancer where treatment requires either inhibition of Notch signaling [86] or treatment with the telomerase inhibitor Imetelstat [87]. These putative biomarkers are summarized in Table 1.

## 4. Putative Breast Cancer Stem Cell ERα(−) Biomarkers

Differentiation is an ongoing process in the human mammary gland, culminating during pregnancy and lactation when numerous lobulo-acinar structures containing milk-secreting alveolar cells are formed through extensive proliferation [88]. The cessation of lactation is accompanied by massive apoptosis and tissue remodeling as the gland reverts to a structure resembling that prior to pregnancy. These processes, which may recur multiple times, require a group of cells with high proliferative potential and differentiation ability, a description that fits the definition of stem cells or early progenitor cells [89]. It has been reported that a functional mammary gland is formed by a single cell and a mutated stem cell could be the common cellular origin of teratocarcinomas and epithelial cancers [90,91]. Electron microscopy studies of rodent mammary epithelium indicate that stem cells are relatively undistinguished “small light cells” that occupy an intermediate position between the ductal lumen and basement membrane [92]. Adult stem cells are slow-dividing, long-lived cells that by their very nature are exposed to damaging agents for long periods of time, resulting in the accumulation of mutations that might eventually lead to their transformation [93]. Stem cell migration is regulated by specific chemokines and their receptors [94], and one of these receptors, CXCR4, was found to be overexpressed in metastatic breast cancer [95], leading to the conclusion that tumorigenic stem cells are not only the origin of primary tumors, but might also be responsible for metastases as the result of tumor cell homing and growth at sites removed from the parent tumor [88,96]. An additional study revealed that human mammary luminal epithelial cells contain the progenitors of myoepithelial cells [97], and therefore, the focal disruptions in myoepithelial cell layers observed in conjunction with DCIS may be the result of a halt in the differentiation of cancerous luminal stem cells, which then fail to form epithelial and myoepithelial cells.

Comparisons of the proteins extracted from normal or cancerous breast tissues are limited by the fact that breast tissues are composed of heterogeneous cell types. The identification of novel markers for human breast cancer stem cells (comprising approximately 2% of the total cells) requires their separation from the rest of the cells (the remaining 98%) contained *in vivo*. It is believed that breast cancer is functionally heterogeneous and that a rare human breast cancer initiating cell (BrCa-IC) is the only cell type capable of establishing human breast cancer after transplant into NOD/SCID mice [93,98]. To find luminal breast stem cell markers, complementary studies have been performed indicating that human breast epithelial stem cells are also located at this intermediate position, and can be characterized by the presence of cytosolic markers such as cytokeratin 19 and cell surface proteins such as epithelial specific antigen (ESA) and stem cell antigen-1 (Sca-1), in addition to the absence of sialomucins (MUC) [99–101]. Four cell surface markers, adhesion molecules CD44 and CD24, the breast/ovarian cancer-specific marker B38.1, and ESA, as well as lineage markers (Lin^+^) for hematopoietic, endothelial, mesothelial, and fibroblast cells, have been used to identify and characterize putative human breast cancer stem cells. Only Lin^−^/ESA^+^/CD44^+^/CD24^−/low^/B38.1^+^ cells generated breast tumors in immunocompromised female mice [102]. Embryonic stem cell marker SOX2 is expressed in early stage breast carcinoma [103]. Breast cancer stem cells were also found to express IL-4, IL-10, and TGF-β1 and upregulate the expression of regulatory molecules on T cells.

## 5. Promising Cytoskeleton Candidates

Structural protein differences between ERα(+) and ERα(−) breast cancer have not been addressed adequately. Here we identify a few promising structural proteins that need to be analyzed to understand the differences in microtubule dynamics between these two types of breast cancer. Microtubule-targeting drugs (MTTD) used to treat breast cancer exercise their anti-mitotic activities by suppressing microtubule dynamics. They are classified into two categories: the first is composed of vinca alkaloids (Vincristine, Vinblastine, *etc*.), which are β-tubulin-binding, microtubule-destabilizing small molecules; and the second is composed of taxanes (Taxol and Docetaxel), which are also β-tubulin-binding, but stabilize microtubules. Many nuclear movements are microtubule-dependent and the cytoskeleton plays a major role in breaking cell symmetry along Microtubules-Associated Proteins (MAPS) and histones [104–107]. The mechanism of nuclear movement to reorient the centrosome in migrating fibroblasts has recently been identified [108]. A model for cleavage plane geometry during mitosis has also been identified [109]. Acting as anchors for nesprin-2G-SUN2 TAN lines, A-type lamins allow productive movement and proper positioning of the nucleus by actin [110]. A recently identified pathway where actin filaments, promoted by VASP, grow transiently from barbed ends [111] and then undergo a catastrophic burst of disassembly is worth investigating in ERα(+) *versus* ERα(−) breast cancer cells [112].

A mechanism of organelle inheritance during mitosis, important in breast cancer, has recently been identified [113]. Along with GRASP Grh1 [114] and BLOC-1, -2, and -3 [115,116], tubulins play a role in organelle biogenesis and modifications to this family of proteins play a major role in directing intracellular trafficking [117,118], microtubule dynamics [119], microtentacle formation [120], epithelial-to-mesenchymal transition [121], and mitotic events [122,123]. FG domains present in tubulin-α8 form a tubular gate structure, or transporter, at the nuclear pore complex center featuring two separate mechanisms directing trafficking [124,125]. The mitochondrial membrane tubulation activity of OPA1 that is suppressed by GTPγS needs to be analyzed in ERα(+) and ERα(−) breast cancer cells [126–128].

Core microtubule-binding complexes at the kinetochore play a major role in coupling force generation to microtubule plus-end polymerization and depolymerization [129–131]. Formins have recently been recognized as prominent regulators of the microtubule (MT) cytoskeleton where they modulate the dynamics of selected MTs during interphase and mitosis [132]. The 9 + 2 axoneme, a microtubule-based machine that powers the oscillatory beating of cilia and flagella, and intraflagellar transport machinery is required for cilia assembly [133,134]. Kinetochore-microtubule dynamics regulate mitotic progression [135,136] and avoid chromosomal missegregations that may lead to aneuploidy, an important phenomenon in breast cancer [137,138]. The level of phostensin, undetectable in metastatic breast cancer, is also good candidate [139]. Nup107–160 complex and gamma-TuRC regulate microtubule polymerization at kinetochores [140]. Mitotic kinesin CENP-E promotes microtubule plus-end elongation and utilizes non-motor microtubule binding sites to tune its microtubule attachment dynamics, enabling it to efficiently align and sort microtubules during metaphase spindle assembly and function [141–143]. Proper organization of microtubule minus-ends is needed for midzone stability, cytokinesis [144], and chromosome segregation [145]. Nucleoporins play a role in early mitotic progression and insure that daughter cells are generated only when fully formed NPCs are present [146,147]. The perinucleolar compartment that forms in cancer cells is highly enriched with a subset of recently characterized polymerase III RNAs and RNA-binding proteins [148,149]. Diseases of the nuclear envelope also need to be analyzed in ERα(+) and ERα(−) breast cancer cells [150]. Non-muscle myosin II plays essential roles in embryonic and post-embryonic development [151]. MyoII and IQGAP/cortexillin play key roles in spatially and temporally regulating leading-edge activity, and RasC activity and the spatiotemporal activation of TORC2 are tightly controlled at the leading edge of chemotaxing cells [152–154]. Vinculin also plays a role in physiological processes such as cell motility, migration, development, and wound healing and loss of this protein has been associated with cancer phenotypes [155], making it another factor that needs to be addressed in ERα(+) and ERα(−) breast cancer. A telomere maintenance mechanism has also been described [156]. A recent finding shows that Rap1 is sufficient to suppress most of the telomere aberrations [157], yet these aberrations in ERα(+) and ERα(−) breast cancer cells have not been addressed. The list of cytoskeleton candidates are summarized in Table 2.

## 6. Conclusions

Estrogen receptors α and β, among other estrogen receptors [158], play a major role in the development of mammary glands. ERα(−) breast cancer remains one of the most therapy-resistant diseases with ERα(−) cancer cells expressing fewer proteins than their ERα(+) counterparts. Cancer stem cells, a minor population of ERα(−) breast tumor cells, retain the stem cell property of self-renewal. Targeting ERα(−) and breast cancer stem cells is necessary to improve ERα(−) breast cancer outcome. Microtubule-targeting drugs have been successful in treating breast cancer despite their lack of cancer specificity. Cytoskeleton candidates that are specific to ERα(−) breast cancer and breast cancer stem cells also need to be identified for future targeting. Cytoskeleton proteins involved in organelle biogenesis, mitosis, perinuclear development, and telomere aberrations need to be analyzed and differences between proteins expressed by ERα(+) and ERα(−) need to be identified in order to specifically target ERα(−) breast cancer.

## Figures and Tables

**Table 1:** Putative estrogen receptor related breast cancer biomarkers.

Putative Biomarkers	ERα(+)	ERα(−)	Function	References
611-CTF	−	+	Resistance to treatment	[59]
FKBPL	+	−	Regulation of ER expression	[61]
BP1	−	+	Cell proliferation	[72]
Superoxide Dismutase	+	−	Anti-oxidant	[73]
Ral A Binding Protein	+	−	Tumorigenesis–Metastasis	[74–75]
Galectin-1	+	−	Apoptosis	[76]
Uridine Phosphorylase 2	+	−	Contributes to drug efficacy	[77]
Cellular retionic acid-binding protein 1	+	−	Cell growth and differentiation	[78]
Protein S100-A11	+	−	Tumorigenesis	[79]
Nucleoside Diphosphate Kinase A	+	−	Metastasis suppressor	[81–82]
Rho GDP-Dissociation inhibitor 1	−	+	Resistance to drugs	[83–85]

**Table 2:** Promising cytoskeleton protein candidates.

Protein Candidate	Function	References
A-type lamins	Proper Positioning of the Nucleus	[110]
VASP	Actin Filament Growth	[111]
GRASP Grh1	Organelle Biogenesis	[114]
BLOC-1, -2, and -3	Organelle Biogenesis	[115–116]
OPA1	Mitochondrial Tubulation	[126–128]
Formins	Microtubule Regulator	[132]
Nup107-160	Microtubule Polymerization	[140]
gamma-TuRC	Microtubule Polymerization	[140]
CENP-E	Spindle Assembly	[141–143]
Nucleoporins	Mitotic Progression	[146–147]
Vinculin	Cell Motility	[155]
Rap1	Suppression of Telomere Aberrations	[157]
